# EEG response and productivity outcome under changing indoor environment

**DOI:** 10.3389/fpubh.2026.1767357

**Published:** 2026-03-05

**Authors:** Shuyao Zhang, Tianyi Ma, Sophia Zuoqiu

**Affiliations:** Sichuan University-Pittsburgh Institute, Chengdu, China

**Keywords:** Concentration Power Index (CPI), electroencephalogram (EEG), Grooved Pegboard Test (GPT), human capital approach (HCA), indoor environment, productivity

## Abstract

**Introduction:**

Indoor environmental quality (IEQ) is a key determinant of health economics and specifically of productivity. In this research, we investigated the effect of three indoor environmental factors (temperature, acoustics, and luminescence) under four different scenarios to generate real-world evidence on how changes in the indoor environment affect productivity-related outcomes.

**Methods:**

Two levels of temperature (15–17 °C and 23–25 °C), two levels of acoustics (45 dB and 85 dB), and two levels of luminescence (300 lx and 700 lx) were tested. We recruited 30 undergraduate students and measured their brain activity using electroencephalogram (EEG) to obtain the Concentration Power Index (CPI) and cognitive response with the Grooved Pegboard Test (GPT). Individual’s productivity was measured objectively by the CPI and subjectively by the GPT. Mixed-effects regression analysis indicated that all three indoor environmental factors have statistically significant effects on productivity.

**Results:**

Relatively higher temperature is associated with increased productivity, as indicated by higher CPI under the experimental conditions. Lower acoustics are associated with productivity as indicated by lower GPT under the conditions investigated. Luminescence is inversely associated with productivity, as higher luminescence has a statistically significant correlation with lower GPT within the tested luminescence range. However, males and females responded differently under the same indoor environment. Specifically, relatively lower temperature is associated with higher productivity for females and lower GPT for males. Higher acoustics and lower luminescence are associated with productivity for males.

**Discussion:**

Overall, the study results suggest that indoor temperature at 23–25 °C, acoustics at 45 dB, and luminescence at 700 lx provide a more favorable and productive environment for young adults. Louder environment and excessive lighting correspond to lower human capital approach (HCA) values and higher temperature corresponds to HCA again. With first-hand experimental results, this study provides real-world indoor environmental data and could provide scientific support for optimizing indoor environments and contributing to the future interior environment design to enhance human productivity.

## 1 Introduction

In modern society, humans are staying indoors more, specifically, people spend about 87% of a day inside the building (1). Accordingly, the indoor environment can be a key factor that influences productivity performance. Indoor environmental quality (IEQ) is a widespread and modifiable factor that affects human health, cognitive abilities and work efficiency, which plays a significant role in actual environments such as offices and schools. The use of real-world data to evaluate IEQ and its impact on productivity has become a significant focus of current research. Indoor environmental factors, such as temperature, acoustics, and luminescence, can affect performance by influencing work comfort and concentration (2). Poor indoor environment has a detrimental effect on human health and work efficiency. An extreme indoor environment can diminish learning efficiency, and even affect students’ learning outcomes (3), and relocating to buildings with relatively improved indoor environmental conditions can decrease the frequency of reporting health problems (4). Moreover, certain ventilation rates and appropriate temperatures can improve students’ health and learning performance (5). Hence it is necessary to improve the quality of the indoor environment. Productivity is closely linked to educational achievement, work performance and attendance, these effects have direct implications for health and economic outcomes at both the individual and population levels. Poor indoor environment quality may contribute to errors and stress-related health problems, which in turn impose costs on employees, students and organizations. Conversely, optimizing indoor environmental conditions represents a relatively low-cost, upstream intervention than can support productivity and potentially improve health-related economic outcomes.

However, there are limitations in current regulations of indoor environments in China. For instance, the regulation of the government suggested that the luminescence for tables and blackboards should be higher than 300 lx and 500 lx (6), the acoustics intensity of the study area should be less than 55 dB in days and 45 dB in nights (7), and the relative humidity in winter indoors should be between 30 and 60% (8). Those regulations limit the range of environmental factors, which can only satisfy the basic requirements of people in the indoor environment, but these lack specifications of what kind of indoor environmental conditions could be conducive to performance. In other words, how are the temperature, acoustics, and luminescence associated with productivity? This research focuses on understanding the specific correlation between indoor parameters and human performance through subjective and objective evaluations of performance to explore the impact of changing indoor environment on productivity.

This research designed four experimental scenarios and modulated temperature, acoustics, and luminescence within each scenario, respectively. The electroencephalogram (EEG) and electrocardiogram (ECG) were recorded in the experiment process. This experiment used the completion time of the Grooved Pegboard Test (GPT) as the subjective outcome and used the Concentration Power Index (CPI) which was obtained from EEG data as an objective outcome. After that, this study analyzed the statistical model of CPI and GPT, explained the effects of different indoor environmental variables on attention, performance, and productivity, and explored how these results differed by gender. This research may provide recommendations for improving indoor environments and inform future interior environment designs to enhance human productivity.

The indoor environment and its impacts have been a topic of wide concern. Many scholars have conducted research on the effects of changing indoor environments. This chapter summarizes the current state of research in three areas: how the indoor environment affects performance and productivity, how the indoor environment affects performance by influencing brain activity, and how GPT represents performance.

Studies have demonstrated that indoor environmental factors can influence people’s cognitive performance. The analysis by Juan and Chen (9) showed that the changes in temperature, acoustics, and luminescence all have a significant influence on the subjects’ concentration. Liu et al. (10) have found that 600 lx in luminescence and 45 dB in acoustics lead to the best reading performance. Another research found that the best performance occurred at 24 °C and 40%RH (11). The experiments by Hugge (12), Meng (13), and Ke et al. (14) found that loud noise reduces the ability of memory and identification. Ru’s et al. (15) experiments about luminescence revealed that both high and low levels of luminescence can speed up the response significantly. Together, these findings suggest that lower acoustics, moderate temperature, moderate relative humidity, and moderate luminescence lead to better performance.

Established studies have also shown that there are differences in preferences for indoor environments by gender. The experiments by Hu et al. (16) suggested that the thermal comfort temperature of males is lower by about 1–2 °C than females. Jing et al. (17) found that males tend to make more mistakes in cognitive tasks under noisy environments, while there is no difference in mistake rates across noise environments for females. According to Kakitsuba’s (18) experiment about gender differences in LED lighting conditions, there is no significant difference in comfort lighting conditions between males and females.

Performance can be evaluated objectively and subjectively. EEG is a quantifiable measurement of brain activity, which can be used as an objective indicator of performance (19, 20). Some scholars have used EEG to explore the effects of changing indoor environments on performance. Deng et al. (21) showed that EEG band power is strongly associated with office productivity under combined indoor air quality and noise conditions. Shan et al. (22) reported that EEG alpha and theta bands correlate with subjective perception and task performance under different indoor air quality conditions. Zhu’s et al. (23) monitored the EEG changes under different temperatures and relative humidities and indicated that too high relative humidity would reduce the power of beta waves and lower the thinking level. Wang et al. (24) found that frontal theta power varies significantly across thermal conditions. Han and Chun (25) indicated that both thermal and cold stimuli significantly modulate theta and beta wave activity. Choi et al. (26) also monitored EEG changes under combined environments including temperature, acoustics, and ventilation, and the result suggested that the poor indoor environment made the occupants more stressed with relatively stronger high-beta waves. Those studies chose to examine the EEG band powers, while the other two groups used the CPI to represent the attention level. The CPI quantifies attention focus levels by computationally analyzing EEG signals, specifically measuring neural oscillation patterns in frequency bands like beta waves that correlate with sustained cognitive engagement. Choi et al. (27) found that extreme temperatures, including too hot and too cold, will diminish the concentration and performance. The research of Li not only pointed out the significant relationship between temperature and CPI but also indicated that there is a positive relationship between CPI and performance index (28).

The GPT is a widely used neuroscience test. According to Skogan et al. (29), shorter completion times indicate greater motor skill and operational efficiency. As a valid assessment tool of cognitive load, GPT performance correlates with autonomic responses (30). GPT’s sensitivity to cognitive load allows it to express the reflections to the psyche during uncomfortable indoor environments. The research of Kanj et al. (31) revealed that GPT completion time is significantly related to the information processing speed and working memory. Johnson and Lesniak-Karpiak (32) indicated that the longer completion time of GPT is related to the reduction of attention, which also means a decrease in performance (33). Thus, the completion time of GPT can represent the level of attention, performance, and productivity.

Previous studies have analyzed the impact of the indoor environment extensively, with some incorporating EEG as the outcome variable. However, studies on the indoor environment rarely evaluated physiological response to changes in indoor environmental parameters using both objective and subjective indicators. Such triangulation by a combination of subjective and objective indicators would verify and enhance the understanding of environmental health relationships. This research is designed to combine GPT, a subjective evaluation, and EEG, an objective evaluation, to comprehensively analyze the effects of the indoor environment on productivity.

Beyond used real world data to optimize indoor environments and enhance human productivity, from a sustainable development perspective, this study contributed to multiple United Nations Sustainable Development Goals (SDGs), particularly the goals of Good Health and Wellbeing, and Decent Work and Economic Growth. Indoor environmental quality is a fundamental determinant of cognitive health, occupational wellbeing, and labor productivity, all of which are essential components of sustainable economic development.

## 2 Methods

### 2.1 Subjects

Thirty undergraduate students (M_age_ = 20.04 ± 1.45 years) from Sichuan University participated in this study, of which 24 are female and six are male. Participants voluntarily signed up for the experiment. The subjects were all healthy and had no visual or hearing impairment except myopia. Participants wore their regular indoor autumn clothing, and the clothing insulation is approximately 0.8clo. All of the subjects participated in the four scenarios of the experiment. This study is reviewed and approved by Institutional Review Board of Sichuan University West China Medical School (IRB number: KS20250686).

### 2.2 Experimental conditions

Experiments were performed between December 2024 and March 2025 on Jiangan Campus, Sichuan University, Chengdu, Sichuan. All experiments were conducted during the daytime on weekdays.

**Figure 1:**
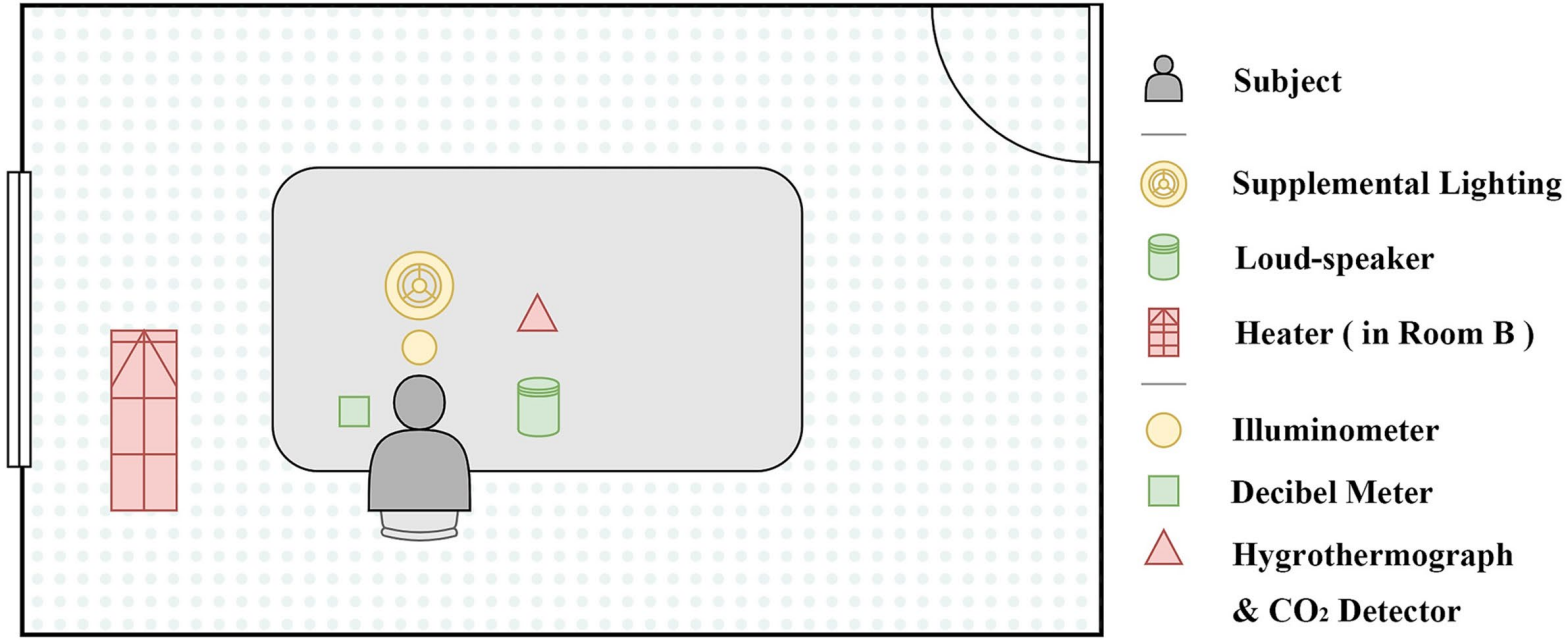
Experiment layout.

**Figure 2:**
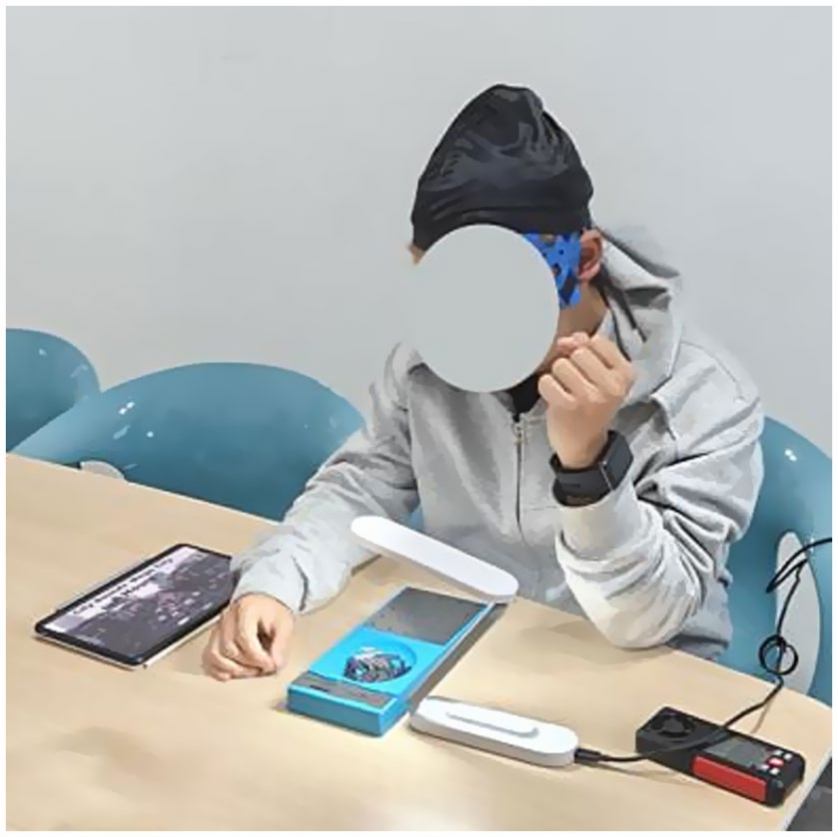
Schematic of the experimental process.

The designed experimental conditions are shown in Table 1. The experiments provided two temperature levels: 15–17 °C and 23–25 °C, two acoustics levels: 45 dB and 85 dB, and two luminescence levels: 300 lx and 700 lx. It formed controlled experiments between experimental scenarios. The temperature of Scenario Cooler was designed to represent a relatively cool but tolerable indoor environment, and exposure duration was limited to the experimental process. For Scenario Standard and Cooler, the temperature changed while acoustics and luminescence remained the same. The controlled experiments were also performed in Scenario Standard and Louder, as well as Scenario Standard and Darker. Other control variables were recorded simultaneously. To avoid interference from outdoor daylight variation, curtains were kept closed and artificial lighting was used as the sole light source during experiments. Figures 1, 2 show the layout of the experiment and the actual record during the experiment below.

**Table 1:** Experimental conditions.

Factors	Scenarios				Method
	Cooler	Standard	Louder	Darker	
Temperature	15–17 °C	23–25 °C	23–25 °C	23–25 °C	Move to another test room with a heater
Acoustics	45 dB	45 dB	85 dB	45 dB	Playing traffic noise audio
Luminescence	700 lx	700 lx	700 lx	300 lx	Supplemental lighting

### 2.3 Experimental procedures

**Figure 3:**
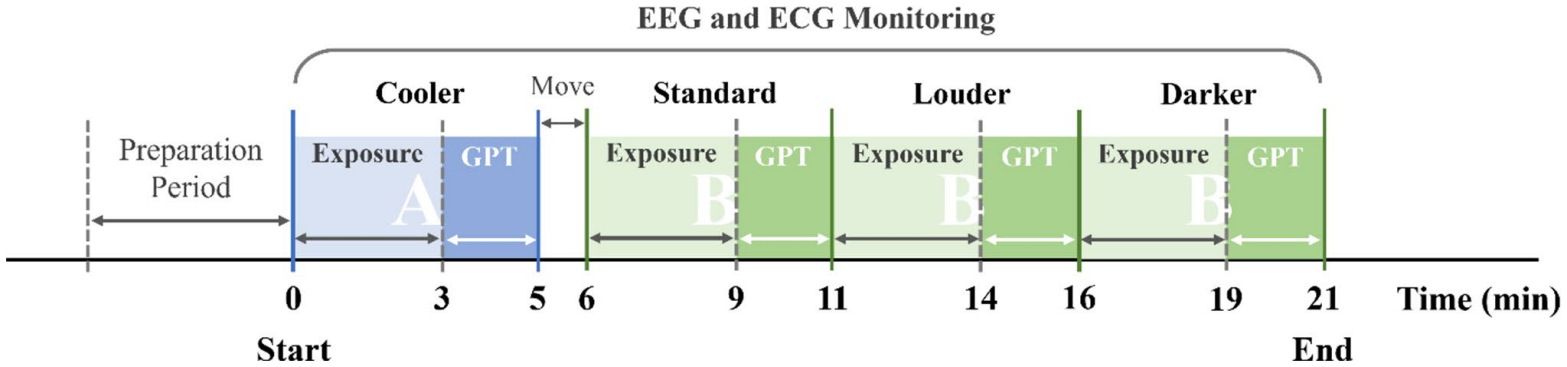
Experimental procedures.

The experiments completed four scenarios in two test rooms. The difference between Room A and Room B was the thermal conditions. In the preparation period, experimenters helped the subject to wear the EEG and ECG devices. At the same time, the subject stayed in Room A at 15–17 °C and relaxed. The baseline EEG was recorded at that time. In the test period, the subjects had 3 min of exposure in each scenario, after that they were asked to complete the GPT. After finishing the experiment of Scenario Cooler, the subject moved to Room B with 23–25 °C to conduct the experiments in other three scenarios. After finishing all four scenarios, the experiment ended and the device stopped recording. Figure 3 presents the experimental procedure.

### 2.4 Measurements

#### 2.4.1 Measurements of physical parameters

Five indoor environmental parameters, temperature, acoustics, luminescence, relative humidity, and CO_2_ concentration, were measured by specialized instruments summarized in Supplementary Table S1. All physical parameters were recorded at the seat of subjects once in each scenario.

#### 2.4.2 Measurements of objective outcome

**Figure 4:**
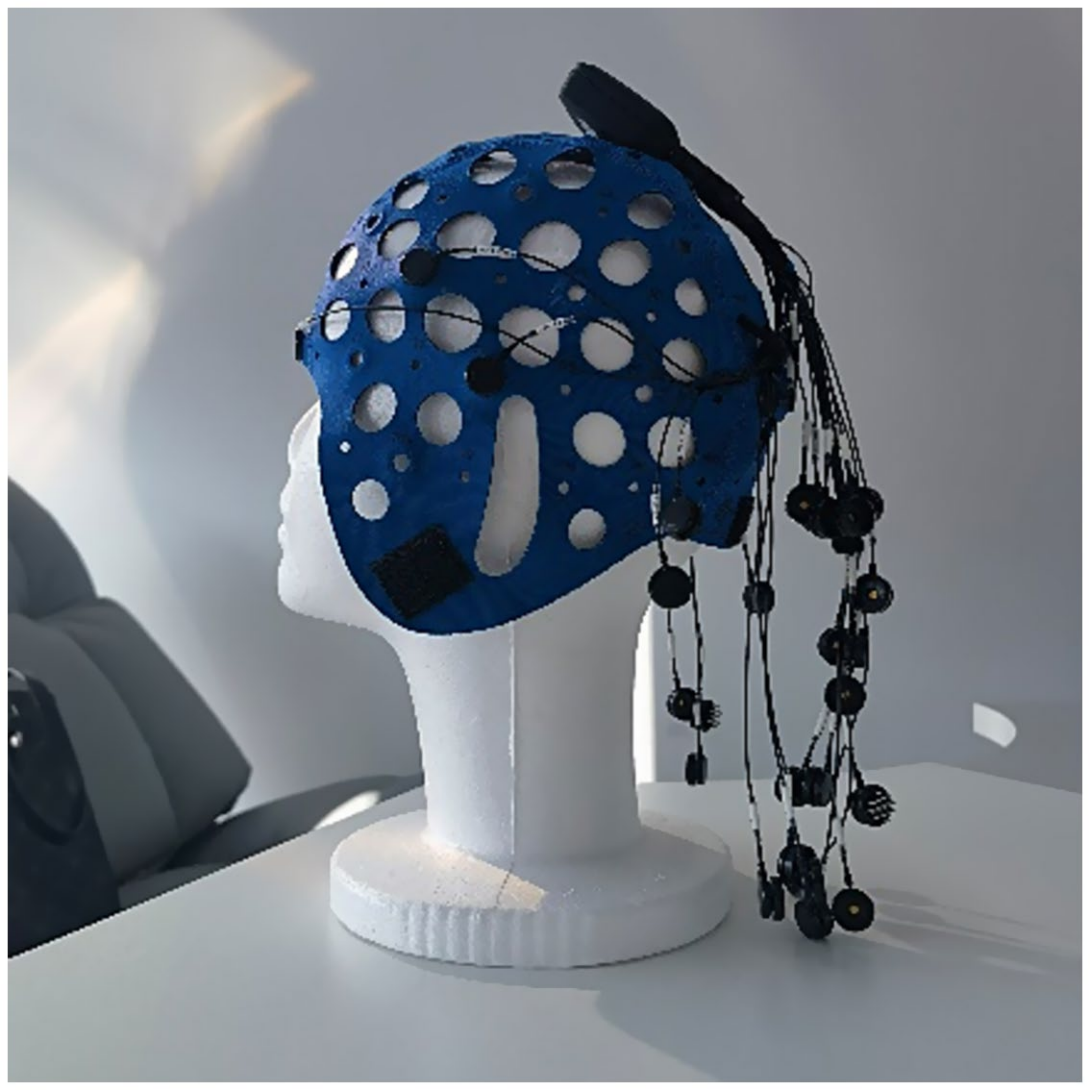
Mentalab Explore+.

**Figure 5:**
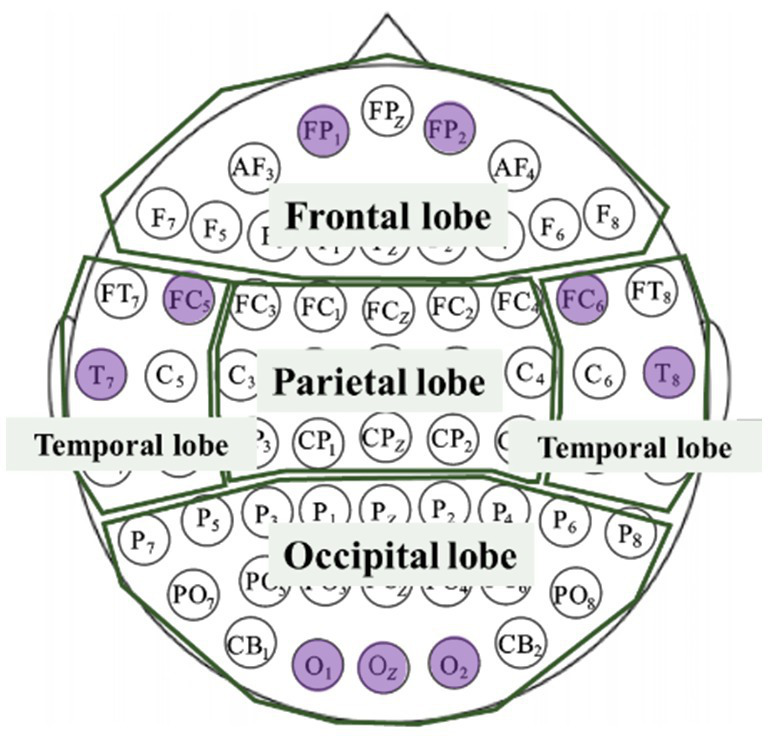
Channels location of EEG.

EEG was measured by the Mentalab Explore+ (Mentalab GmbH, Munich, Germany) as shown in Figure 4, which has been verified as a reliable and effective EEG recording device (34) and has been used in assessing cognitive and attention previously (35). Mentalab Explore+ features 32 selectable EEG channels and a reference channel at M2. The 9 channels are located at FP1, FC5, T7, O1, Oz, O2, T8, FC6, and FP2 as shown in Figure 5. They cover the three main parts of head including Frontal lobe, Temporal lobe, and Occipital lobe. This research used dry electrodes and the sampling frequency was 250 Hz. The signals measured by Mentalab Explore+ were recorded by the supporting software called Explore Desktop. This software enables real-time viewing of sensor information and saves the EEG data for later processing. The data recorded were loaded into MATLAB to extract EEG features.

ECG information including heart rate and pulse pressure were measured using HUAWEI WATCH D (Huawei Technologies Co., Ltd., China). It is a medical device in the form of intelligent bracelets which has secured regulatory approval by the National Medical Products Administration of China (Registration No. 20212071428) that allow for heart rate monitoring and blood pressure measurement. Previous research has demonstrated that the accuracy of this device was validated according to the requirements of Advancement of Medical Instrumentation/European Society of Hypertension/ International Organization for Standardization Universal Standard (36), and the measurements provided by the device showed good agreement with clinical sphygmomanometers (37). The heart rate and blood pressure of each subject in each scenario were measured by the mean heart rate and mean blood pressure of them finishing a single GPT. The pulse pressure is calculated by the difference between systolic pressure and diastolic pressure.

#### 2.4.3 Measurements of subjective outcome

**Figure 6:**
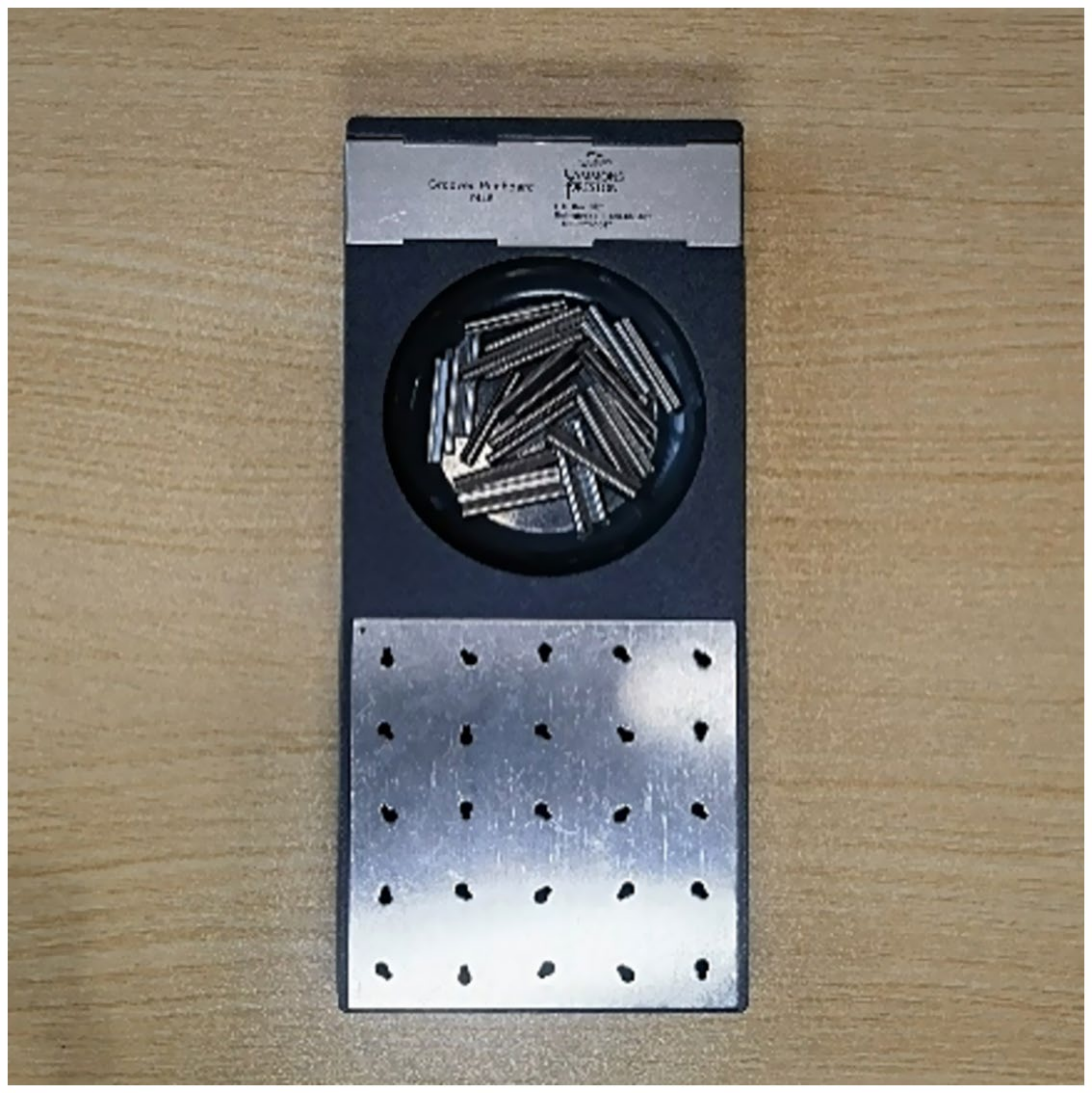
Grooved pegboard.

**Figure 7:**
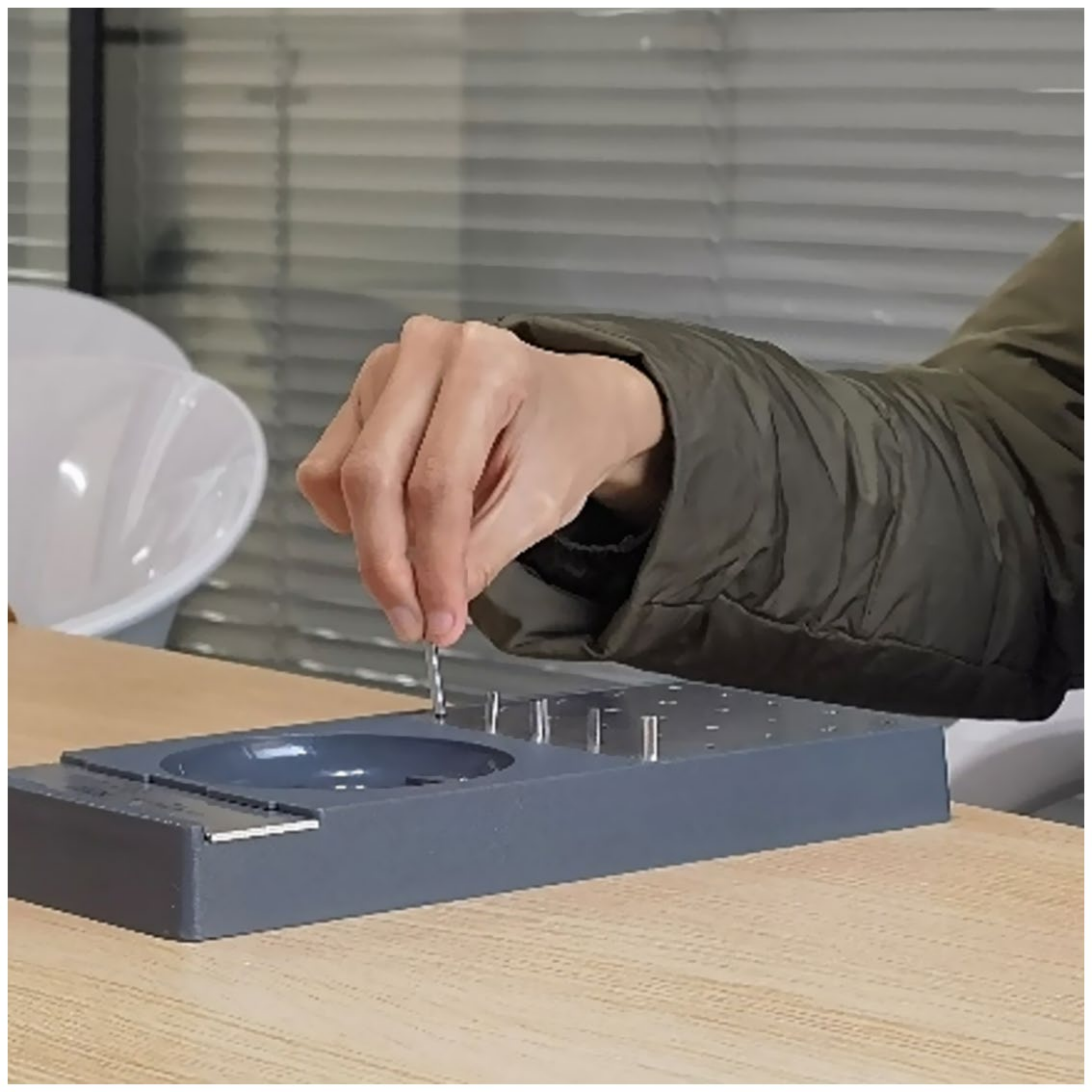
Picture of subjects completing the GPT.

The subjective outcome of productivity was measured by the completion time of the GPT (38) as shown in Figures 6, 7. The GPT is a standardized test to evaluate manual dexterity, swift visual-motor coordination, and psychomotor speed (39). It is widely used in research in the field of neuroscience. Previous research indicated that the performance of GPT is related to attention (40) and cognitive functions (41). According to Streng, there is a relationship between performance on GPT and tests of attention (42). This research used the GPT performance to measure the attention and performance of subjects. The pegboard used in this research was the Grooved Pegboard 7446 from Sammons Preston Inc., which has 25 holes with randomly positioned slot. Pegs with keys should be inserted accurately into the hole after rotation. This research recorded the time of using subjects dominate hands to fill all the 25 holes of the grooved pegboard. The subjects began with the GPT after the experimenter’s word of command. The timing by the experimenter ceased upon the completion of the last hole in the pegboard, at which point the elapsed time was recorded as the subject’s duration for the GPT.

### 2.5 EEG data preprocessing and processing

#### 2.5.1 EEG data preprocessing

EEG preprocessing was conducted by the EEGLAB toolbox (version 2024.2.1) in MATLAB (R2022a) environment (43). The raw EEG data were imported via the supporting plug-in of Mentalab. Electrode positioning was performed first. The frequency was filtered between 1 and 30 Hz, and the notch filter was performed at 50 Hz. Baseline corrections for test time periods were performed, and bad periods were removed. The bad electrodes were removed and interpolated spherically. Independent component analysis (ICA) removed artifacts like eye blink, eye movement, muscle activities, heart activities, and line noise.

#### 2.5.2 EEG data processing

Using the FieldTrip toolbox in MATLAB environment (44), applied Fourier Transformation to the preprocessed EEG data to convert time-domain features into frequency-domain features, and extracted the band powers. The EEG band powers were extracted in three following frequencies: SMR (12–15 Hz), Mid Beta (15–20 Hz), and Theta (4–8 Hz). The power ratio CPI was used to measure subjects’ attention levels (45). Sensorimotor rhythm (SMR) waves are frequency waves in 12–15 Hz, which cover both alpha (8–13 Hz) and beta (13–30 Hz) waves related to human attention (46). Mid beta waves at the frequency of 15–20 Hz also represent concentration and brain activities like memory and calculation (27). Theta waves at a frequency of 4–8 Hz relate to sleep and deep spirit. The relationship between CPI and indoor thermal conditions has been studied in previous research (28). The CPI is calculated by Equation (1):


$$\mathrm{CPI}=\frac{\;(\mathrm{SMR}+\mathrm{Mid}\;\text{Beta})}{\text{Theta}}$$ (1)

### 2.6 Statistical analysis

This study measures productivity using two explained variables, which are the CPI representing the objective outcome and GPT representing the subjective outcome. The main explanatory variables are temperature, acoustics, and luminescence. Relative humidity, CO_2_ concentration, heart rate, pulse pressure, and number of tests were included as control variables to account for background environmental conditions and individual physical states.

This research uses the mixed-effects regression model to evaluate the influence of the indoor environment on the CPI and GPT while accounting for individual differences between study subjects. The associations between indoor environmental factors and productivity outcomes reported in this study were derived from the estimated coefficients of the mixed-effects regression models. All statistical analyses were performed using Stata 18.0 (Stata Corp., Texas, USA). Statistical significance was assessed using two-sided tests with a significance level of *p* < 0.05. The model is constructed by Equation (2):


$$Y_{\mathrm{ij}}=\beta_{0}+\beta_{1\sim 5}x_{1\sim 5j}+\beta_{6\sim 7}x_{6\sim 7j}+\beta_{8}x_{8j}+\varepsilon \;$$ (2)

Where $Y_{\mathrm{ij}}$ : The CPI or GPT result for subject j , $x_{1\sim 5j}$ : The value of environmental factors including temperature, acoustics, luminescence, relative humidity, and CO_2_ concentration for subject j , $x_{6\sim 7j}$ : The value of physiological factors including heart rate and pulse pressure for subject $j,x_{8j}$ : The number of tests for subject j , ε : The residual of the model.

### 2.7 Health economic calculation

Human capital approach (HCA) assesses productivity changes by multiplying output ratio variations with estimated individual wages, and wage estimates serve as a proxy for marginal productivity (47). For instance, the calculation of HCA often follows the formula: absent rate (or presenteeism rate) × wage (48). In this study, CPI and GPT represent the objective and subjective measures of productivity, respectively. Based on this principle, the presenteeism rate is calculated as the ratio of CPI or GPT change rates to their baseline values Base . The product of this presenteeism rate and average wages represents the monetary value of productivity changes induced by indoor environments. Since GPT is negatively correlated with productivity, the sign is reversed in the $\mathrm{HC}A_{\mathrm{GPT}}$ formula. The HCA is calculated as follows Equations (3–4):


$$\mathrm{HC}A_{\mathrm{CPI}}=\frac{\beta_{1\sim 3}\_\mathrm{CPI}}{\text{Base}\_\mathrm{CPI}}\times \text{Annual Wage}$$ (3)

$$\mathrm{HC}A_{\mathrm{GPT}}=-\frac{\beta_{1\sim 3\_\mathrm{GPT}}}{\text{Base}\_\mathrm{GPT}}\times \text{Annual Wage}$$ (4)

The value of Base is the mean of CPI or GPT for all subjects under Scenario Standard. The Annual Wage represents the median annual wage of scientific researchers in Sichuan Province China in 2024 (49), measured in RMB. Annual Wage = ¥90,000.

## 3 Results

### 3.1 Descriptive statistics

Descriptive statistics of all variables are provided in Table 4. To show the result of descriptive statistics intuitively, the environmental parameters of the four scenarios can be summarized in a radar chart as shown in Supplementary Figure S1.

The descriptive statistics show that the targeted temperature, acoustics, and luminescence were reached. The temperature was controlled within ±0.9 °C, the acoustics deviation did not exceed 3 dB, and the luminescence was not changed over 12 lx.

### 3.2 The effects of indoor environment on productivity outcome

Table 2 presents the estimation results for the mixed effects model. In addition to the primary environmental factors, including Temperature, Acoustics, and Luminescence, correlations between the outcomes and control variables were examined to assess potential confounding influences. The values presented in Tables 3, 4 represent regression coefficients ( β ) estimated from the mixed-effects model.

**Table 2:** Results of indoor environmental factors impacting CPI and GPT.

Variable	CPI		GPT	
Temperature	0.058*	(0.012, 0.105)	0.209	(−0.201, 0.619)
Acoustics	0.002	(−0.006, 0.010)	0.204***	(0.122, 0.285)
Luminescence	−0.080	(−0.236, 0.078)	−3.501***	(−5.062, −1.940)
Relative humidity	0.014**	(0.004, 0.024)	0.107*	(0.006, 0.208)
CO_2_ concentration	0.172***	(0.111, 0.232)	0.671**	(0.181, 1.160)
Heart rate	0.002	(−0.001, 0.004)	−0.132***	(−0.176, −0.088)
Pulse pressure	0.006*	(0.001, 0.012)	0.129***	(0.064, 0.193)
Number of tests	−0.150	(−0.476, 0.177)	−8.915***	(−12.200, −5.633)

^*^95% confidence interval in parentheses; *p < 0.05, **p < 0.01, ***p < 0.001.

**Table 3:** Summary of regression results from stratified analysis.

Variable	CPI		GPT	
	Female	Male	Female	Male
Temperature	−0.048** (−0.079, −0.017)	0.742*** (0.548, 0.935)	0.200 (−0.219, 0.618)	2.448*** (1.610, 3.285)
Acoustics	−0.013*** (−0.020, −0.006)	0.081*** (0.055, 0.106)	0.274*** (0.187, 0.362)	0.343*** (0.198, 0.488)
Luminescence	0.241*** (0.112, 0.369)	−1.881*** (−2.429, −1.332)	−5.145*** (−6.808, −3.481)	−5.285*** (−8.153, −2.416)
Relative humidity	0.005 (−0.002, 0.012)	0.150*** (0.105, 0.194)	0.187*** (0.087, 0.288)	0.911*** (0.654, 1.168)
CO_2_ concentration	0.076*** (0.039, 0.112)	0.509*** (0.371, 0.647)	−1.210*** (−1.648, −0.772)	7.061*** (6.442, 7.679)
Heart rate	−0.004*** (−0.006, −0.002)	0.027*** (0.016, 0.038)	−0.086*** (−0.121, −0.051)	−0.592*** (−0.649, −0.534)
Pulse pressure	0.002 (−0.002, 0.005)	0.014 (−0.014, 0.041)	0.054 (−0.010, 0.117)	−0.281* (−0.496, −0.065)
Number of tests	0.543*** (0.292, 0.794)	−3.951*** (−5.119, −2.782)	−10.870*** (−14.290, −7.454)	−14.370*** (−20.470, −8.263)

*95% confidence interval in parentheses; *p < 0.05, **p < 0.01, ***p < 0.001.

**Table 4:** Descriptive statistics of variables.

Variable	Cooler		Standard		Louder		Darker	
	Targeted	Monitored	Targeted	Monitored	Targeted	Monitored	Targeted	Monitored
Temperature (°C)	15–17	16.67 ± 0.61	23–25	23.75 ± 0.80	23–25	23.99 ± 0.84	23–25	24.19 ± 0.76
Acoustics (dB)	45	43.34 ± 2.88	45	46.80 ± 1.73	85	85.18 ± 1.62	45	46.83 ± 1.75
Luminescence (100 lx)	7	7.02 ± 0.08	7	6.99 ± 0.09	7	7.02 ± 0.11	3	3.00 ± 0.09
Relative humidity (%)	–	47.23 ± 10.45	–	46.71 ± 10.04	–	41.90 ± 8.28	-	38.93 ± 7.73
CO_2_ concentration (100 ppm)	–	7.62 ± 1.00	–	6.99 ± 0.53	–	6.98 ± 0.41	–	7.51 ± 0.39
Heart rate (bpm)	–	93.70 ± 20.16	–	89.30 ± 18.33	–	93.70 ± 17.87	–	90.10 ± 16.16
Pulse pressure (mmHg)	–	37.70 ± 10.02	–	35.40 ± 8.97	–	35.63 ± 8.22	–	34.43 ± 8.00
CPI	–	0.40 ± 0.72	–	0.54 ± 1.02	–	0.42 ± 0.47	–	0.55 ± 0.61
GPT	–	70.19 ± 11.02	–	63.32 ± 10.38	–	61.14 ± 8.88	–	58.86 ± 8.84

Analyses on CPI disclose that the temperature has a significant positive correlation with CPI at 5% statistical level, hence temperature can significantly promote concentration. Acoustics and luminescence have no significant influence on CPI. The within-group variance accounts for 31.45% of the total variance, indicating that the control of individual differences can explain 31.45% of the total variance. The model of GPT reveals no significant effect of temperature, but it is significant relative to acoustics and luminescence at 0.01 significance level. A higher level of acoustics significantly slows the speed of GPT, while a higher luminescence level significantly speeds up the finish of GPT. The within-group variance makes up 30.57% of the total variance, suggesting that the control of individual differences can explain 30.57% of the total variance.

The observed associations involving control variables were not interpreted as primary findings, but rather as contextual information supporting the robustness of the main analyses. CO_2_ concentration and pulse pressure have a positive correlation on CPI, while other control variables are not statistically significant. Relative humidity, CO_2_ concentration, and pulse pressure have a positive correlation on GPT, while heart rate and number of tests have a negative influence on GPT significantly.

Correlation between variables and the multicollinearity between main explanatory variables were examined prior to conducting regression analysis as shown in Supplementary Table S2. The assessment results indicate that temperature, acoustics, and luminescence are independent of each other (all VIF < 5).

Residual analysis was conducted for both CPI and GPT models. The residual plots in Supplementary Figure S2 show that the residuals of both models are normally distributed, which indicates that the models are reliable.

### 3.3 Stratified analysis by gender

Due to natural physiological and psychological differences between genders, females and males may have different feelings of comfort in the same indoor environment, and the difference leads to different levels of attention and productivity performance. To examine the influence of the changing indoor environment on the productivity for each gender separately, this research divided the entire sample into female (*N* = 24) and male (*N* = 6), and estimated the effects of indoor environmental factors on CPI and GPT, respectively. The results from stratified analysis are presented in Table 3.

The model of CPI reveals that the temperature is positively associated with CPI for females and negatively associated CPI for males at 0.01 significance level. The acoustics has statistically significant association negatively with CPI for females and positively for males. The coefficients of luminescence are significantly positive to CPI for females and significantly negative for males. The model of GPT discloses that the temperature has no statistical significance to GPT for females, while has a positive correlation with GPT significantly for males. For both females and males, acoustics has positive association with GPT significantly, and luminescence has negative correlation with GPT significantly. The result can also be expressed intuitively by Supplementary Figure S3.

### 3.4 Health economics outcome

Calculations were performed using the three primary control variables in Table 2 that exhibit significant results, which are Temperature-CPI, Acoustics-GPT, and Luminescence-GPT. The mean values of CPI and GPT under Scenario Standard were calculated to yield that. $\mathrm{Bas}e_{\mathrm{CPI}}$ = 0.473 and $\mathrm{Bas}e_{\mathrm{GPT}}$ = 63.123. The HCA values corresponding to temperature, acoustics, and luminescence are presented in Table 5.

**Table 5:** Summary of the HCA values.

Variable	HCA
Temperature	11033.39
Acoustics	−290.86
Luminescence	−4991.71

## 4 Discussion

Present results show that the indoor environment has a significant correlation with productivity. Productivity in this study was measured by CPI objectively and the completion time of GPT subjectively. Experiments of this research mainly controlled three indoor environment factors, namely temperature, acoustics, and luminescence, these three factors provide directly applicable evidence on how modest changes in IEQ may influence real-world learning and working performance. This perspective is consistent with viewing IEQ as a modifiable, real-world determinant of productivity and related health outcomes. The regression results show that temperature is significantly and positively associated with CPI, while the correlation between temperature and GPT is not significant. Indoor acoustics also has a significantly positive correlation with GPT, and the effect of acoustics on CPI has no statistical significance. Significant correlation is observed between luminescence and GPT, and there is no significant correlation between luminescence and CPI. The experimental results indicate that under the conditions investigated, the increase in temperature can promote productivity in the objective outcome, the increase in acoustics can inhibit productivity in the subjective outcome, and the increase in luminescence can enhance productivity also in the subjective outcome.

Temperature positively associates CPI at 0.01 significance level, and there is no significant correlation between temperature and GPT. An increase of 1 °C in temperature results in an increase of 0.058 in CPI. The previous study observed that temperature and productivity showed a u-shaped relationship, which means that productivity rises first, and then lowers with the increase of temperature (11, 50). Due to the limitation of experimental conditions, this research only tested a narrow range of temperature comfort to the human body. Therefore, the temperature and CPI in this study show a linear positive correlation. Significant differences emerge between the test results of females and males. For objective outcomes, temperature positively associates CPI for males at 0.01 significance level, which is like the result of the main regression. An increase of 1 °C in temperature results in an increase of 0.742 in CPI for males. The temperature demonstrates a negative correlation on CPI for females significantly. An increase of 1 °C in temperature results in a decrease of 0.048 in CPI for females. The result is consistent with the research of Abbasi et al. (51), which found that females’ comfort and performance measured by alpha and beta waves decreased when temperature increased from 18 °C to 30 °C. For subjective outcomes, the regression result of females shows no significance, but temperature positively associates the GPT for males. An increase of 1 °C in temperature results in an increase of 2.448 s in GPT for males. It might be because males tend to be warmer and feel more uncomfortable under high temperatures (52), and previous research also suggested that the performance was reduced in temperatures higher than 23–24 °C (53).

Acoustics has no significant association with CPI, while it positively associates GPT at 0.01 significance level. This discrepancy may reflect differences in sensitivity between neurophysiological and behavioral measures, and it may also relate to the limited acoustics range examined in this experiment. An increase of 1 dB in acoustics results in an increase of 0.204 s in GPT. The result indicates the increase in acoustics, slower the speed of GPT and decreased productivity. This result agrees with previous studies (13). The association of acoustics with GPT is not influenced by gender. An increase of 1 dB in acoustics results in an increase of 0.274 s for females and 0.343 s for males in GPT. For objective outcomes, significant differences emerge between females and males. An increase of 1 dB in acoustics results in a decrease of 0.013 s in CPI for females, and results in a decrease of 0.081 s for males. The abnormal correlation between acoustics and CPI for males might be because men are less sensitive to noise (54). So, the higher-level acoustics did not reduce CPI in male in this experiment.

Luminescence has no significant association with CPI and a significant negative association with GPT. An increase of 100 lx in luminescence is associated with a decrease of 3.501 s in GPT within the tested luminescence range. Similar results were obtained in previous research (10). The association between luminescence and GPT is similar for different genders. An increase of 100 lx in luminescence results in a decrease of 5.145 s for females and 5.285 s for males in GPT. For objective outcome, an increase of 100 lx in luminescence results in an increase of 0.241 s in CPI for females, and results in a decrease of 1.881 in CPI for males. Previous studies disclosed that people perform better in 700 lx compared to 300 lx (15), which is inconsistent with the results of males. This may be due to the relatively small sample size of males in this experiment and the different sensitivities of individuals to luminescence.

The within-group variance of the main regression is 31.45% for CPI and 30.57% for GPT. This suggests that there is still more than half of the variance not controlled. These insufficiencies are worth continuing to explore in future investigation.

The economic implications of productivity changes under changing indoor environmental conditions were quantified by HCA analysis. Estimated based on the CPI, an increase of 1 °C in temperature results in an annual economic benefit of ¥11033.39 per worker. Estimated by GPT, the effect of an increase of 1 dB in acoustics translating into an annual loss of ¥290.86 per worker. Also, an increase of 100 lx in luminescence results in an annual loss of ¥4991.71 per worker. Those results highlight the economic significance of optimizing indoor economics. Estimations using HCA indicate that relatively minor physiological or cognitive changes can trigger significant shifts in productivity after accumulated over time. By linking real-world cognitive performance data to economic valuation based on productivity, the HCA analysis enhanced the relevance of the findings for real-world decision-making in health economics.

The present study offers several notable strengths. It utilized both subjective and objective evaluation of performance, using the CPI which was obtained from EEG data as an objective outcome alongside the Grooved Pegboard Test as a subjective outcome. This dual-modality approach provides us with a better understanding of how productivity and human activity are affected by indoor environment quality, while the previous study relied on a single metric (55). Moreover, the crossover design within the experiment is a key strength: each participant experienced all four scenarios with different indoor environment quality parameters that would compare with each other in pairs. This design of the experiment helps control individual differences, while many previous studies did not include self-comparisons (31). In summary, these features of the methodology strengthen the confidence of our study and EEG monitoring provides a different view of how indoor environment quality affects human activities.

Despite these strengths, this research still has several limitations. First, the sample was relatively small and homogeneous—30 undergraduate students from Sichuan University—which limits the potential to apply the outcome of our study to broader populations or different age groups. The gender imbalance in the sample (only six males versus 24 females) further limits representativeness, as responses to environmental conditions might differ by gender. Although stratified analysis divided by gender was conducted using mixed-effects models, the relatively small number of male participants may reduce the statistical stability of gender-stratified analysis. Therefore, the observed gender differences observed in this study require further validation and broader application through future research with more balanced gender distribution and larger sample sizes. Additionally, the study did not collect data on perceived temperature or subjective thermal comfort. This means that it cannot be confirmed whether the objectively recorded temperature corresponds to the thermal experience of each participant, thus the factors that could influence concentration and comfort levels might be the same for each participant. Finally, there were unaccounted confounding factors such as individual sensitivity to lighting or background noise. These personal differences were not explicitly controlled and could have affected both productivity outcomes and EEG responses. In general, these limitations suggest that while the findings provide valuable insights into how indoor environment quality affects productivity and human activities, they should be interpreted with caution, and future research with more diverse samples and additional controls should be applied to reinforce and extend these results.

## 5 Conclusion

This study aims to identify the influence of changing indoor environment on productivity. The experiments tested the effect of three indoor environmental factors, which are temperature, acoustics, and luminescence, on productivity by designing four experiments that were controlled against each other. Productivity was measured objectively by CPI and subjectively by GPT. Results from 30 undergraduate participants show that a temperature of 23–25 °C, acoustics at 45 dB, and luminescence at 700 lx associated with higher productivity indicators, with significant effects observed on both objective EEG measures and behavioral test outcomes among the tested conditions.

A relatively higher temperature in the range of 15–25 °C is conducive to the productivity manifested in higher CPI. However, the higher temperature displays negative associates on productivity manifested in lower CPI for females and higher GPT for males. Lower acoustics is associated with higher productivity, which is demonstrated by lower GPT. In contrast, regression results on CPI reveal that men may also perform better in high acoustics environments. Higher luminescence is advantageous for productivity which is reflected in lower GPT under the experimental conditions examined. Nevertheless, regression results indicate that higher productivity may occur at lower luminescence for males which is shown in higher CPI.

In summary, the finding indicate that, under the experimental conditions examined, higher productivity was observed at a temperature at 23–25 °C, acoustics at 45 dB, and luminescence at 700 lx. For different genders, the subjective and objective outcomes are somewhat different. Examining the reasons for these differences, as well as controlling bias due to individual effects are necessary in further studies.

## Data Availability

Raw data supporting the conclusions of this article will be made available by the authors upon request.

## References

[1] Klepeis NE Nelson WC Ott WR Robinson JP Tsang AM Switzer P . The National Human Activity Pattern Survey (NHAPS): a resource for assessing exposure to environmental pollutants. J Expo Sci Environ Epidemiol. (2001) 11:231–52. doi: 10.1038/sj.jea.7500165, 11477521

[2] Roelofsen P. The impact of office environments on employee performance: the design of the workplace as a strategy for productivity enhancement. J Facil Manage. (2002) 1:247–64. doi: 10.1108/14725960310807944

[3] Toyinbo O Shaughnessy R Turunen M Putus T Metsämuuronen J Kurnitski J . Building characteristics, indoor environmental quality, and mathematics achievement in Finnish elementary schools. Build Environ. (2016) 104:114–21. doi: 10.1016/j.buildenv.2016.04.030

[4] MacNaughton P Spengler J Vallarino J Santanam S Satish U Allen J. Environmental perceptions and health before and after relocation to a green building. Build Environ. (2016) 104:138–44. doi: 10.1016/j.buildenv.2016.05.011, 27713594 PMC5047435

[5] Haverinen-Shaughnessy U Shaughnessy RJ Cole EC Toyinbo O Moschandreas DJ. An assessment of indoor environmental quality in schools and its association with health and performance. Build Environ. (2015) 93:35–40. doi: 10.1016/j.buildenv.2015.03.006

[6] National Health Commission of the People’s Republic of China, Standardization Administration of China. (2008).

[7] Ministry of Ecology and Environment of the People’s Republic of China, General Administration of Quality Supervision, Inspection and Quarantine of the People’s Republic of China. (2008).

[8] State Administration for Market Regulation, Standardization Administration of China. (2022).

[9] Juan Y-K Chen Y. The influence of indoor environmental factors on learning: an experiment combining physiological and psychological measurements. Build Environ. (2022) 221:109299. doi: 10.1016/j.buildenv.2022.109299

[10] Liu T Lin C-C Huang K-C Chen Y-C. Effects of noise type, noise intensity, and illumination intensity on reading performance. Appl Acoust. (2017) 120:70–4. doi: 10.1016/j.apacoust.2017.01.019

[11] Liu C Zhang Y Sun L Gao W Jing X Ye W. Influence of indoor air temperature and relative humidity on learning performance of undergraduates. Case Stud Therm Eng. (2021) 28:101458. doi: 10.1016/j.csite.2021.101458

[12] Hygge S. Classroom experiments on the effects of different noise sources and sound levels on long-term recall and recognition in children. Appl Cogn Psychol. (2003) 17:895–914. doi: 10.1002/acp.926

[13] Meng Q An Y Yang D. Effects of acoustic environment on design work performance based on multitask visual cognitive performance in office space. Build Environ. (2021) 205:108296. doi: 10.1016/j.buildenv.2021.108296

[14] Ke J Du J Luo X. The effect of noise content and level on cognitive performance measured by electroencephalography (EEG). Autom Constr. (2021) 130:103836. doi: 10.1016/j.autcon.2021.103836

[15] Ru T Smolders K Chen Q Zhou G De Kort Y. Diurnal effects of illuminance on performance: exploring the moderating role of cognitive domain and task difficulty. Light Res Technol. (2021) 53:727–47. doi: 10.1177/1477153521990645

[16] Hu J He Y Hao X Li N Su Y Qu H. Optimal temperature ranges considering gender differences in thermal comfort, work performance, and sick building syndrome: a winter field study in university classrooms. Energ Buildings. (2022) 254:111554. doi: 10.1016/j.enbuild.2021.111554

[17] Jing Y Jing S Huajian C Chuangang S Yan L. "The gender difference in distraction of background music and noise on the cognitive task performance" In: Proceedings of the 2012 8th International Conference on Natural Computation. Chongqing, Sichuan: IEEE (2012). 584–7.

[18] Kakitsuba N. Comfortable indoor lighting conditions for LEDlights evaluated from psychological and physiological responses. Appl Ergon. (2020) 82:102941. doi: 10.1016/j.apergo.2019.102941, 31505313

[19] Karakas T Yildiz D. Exploring the influence of the built environment on human experience through a neuroscience approach: a systematic review. Front Archit Res. (2020) 9:236–47. doi: 10.1016/j.foar.2019.10.005

[20] Deng Z Dong B Guo X Zhang J. Impact of indoor air quality and multi-domain factors on human productivity and physiological responses: a comprehensive review. Indoor Air. (2024) 2024:5584960. doi: 10.1155/2024/5584960

[21] Deng Z Dong B Guo X Wang X Zhang J. Assessing multi-domain impact of IAQ and noise on productivity with portable air cleaners through physiological signals. Build Environ. (2024) 254:111375. doi: 10.1016/j.buildenv.2024.111375

[22] Shan X Yang E-H Zhou J Chang VWC. Neural-signal electroencephalogram (EEG) methods to improve human-building interaction under different indoor air quality. Energ Build. (2019) 197:188–95. doi: 10.1016/j.enbuild.2019.05.055

[23] Zhu M Liu W Wargocki P. Changes in EEG signals during the cognitive activity at varying air temperature and relative humidity. J Expo Sci Environ Epidemiol. (2020) i:285–98. doi: 10.1038/s41370-019-0154-1, 31235789

[24] Wang X Li D Menassa CC Kamat VR Student M. Investigating the neurophysiological effect of thermal environment on individuals’ performance using electroencephalogram. Comput Civ Eng. (2019) 1:075001. doi: 10.1061/9780784482438.075

[25] Han J Chun C. Differences between EEG during thermal discomfort and thermal displeasure. Build Environ. (2021) 204:108220. doi: 10.1016/j.buildenv.2021.108220

[26] Choi Y Kim M Chun C. Measurement of occupants’ stress based on electroencephalograms (EEG) in twelve combined environments. Build Environ. (2015) 88:65–72. doi: 10.1016/j.buildenv.2014.10.003

[27] Choi Y Kim M Chun C. Effect of temperature on attention ability based on electroencephalogram measurements. Build Environ. (2019) 147:299–304. doi: 10.1016/j.buildenv.2018.10.020

[28] Li Y Li S Gao W Xu W Xu Y Wang J. Exploring the effects of indoor temperature on college students’ physiological responses, cognitive performance and a concentration index derived from EEG signals. Dev Built Environ. (2022) 12:100095. doi: 10.1016/j.dibe.2022.100095

[29] Skogan AH Oerbeck B Christiansen C Lande HL Egeland J. Updated developmental norms for fine motor functions as measured by finger tapping speed and the grooved pegboard test. Dev Neuropsychol. (2018) 43:551–65. doi: 10.1080/87565641.2018.1495724, 30156884

[30] Erdodi LA Kirsch NL Sabelli AG Abeare CA. The grooved pegboard test as a validity indicator—a study on psychogenic interference as a confound in performance validity research. Psychol Inj Law. (2018) 11:307–24. doi: 10.1007/s12207-018-9337-7

[31] Kanj R Zeinoun P Roukoz C Mashmoushi R. Factors associated with motor dexterity on the grooved pegboard test in a Lebanese sample. Appl Neuropsychol Child. (2022) 11:178–83. doi: 10.1080/21622965.2020.1773269 32538205

[32] Johnson JL Lesniak-Karpiak K. The effect of warning on malingering on memory and motor tasks in college samples. Arch Clin Neuropsychol. (1997) 12:231–8. doi: 10.1016/S0887-6177(96)00040-6 14588415

[33] Tolle KA Rahman-Filipiak AM Hale AC Kitchen Andren KA Spencer RJ. Grooved pegboard test as a measure of executive functioning. Appl Neuropsychol Adult. (2020) 27:414–20. doi: 10.1080/23279095.2018.1559165, 30734576

[34] Dirik HB Darendeli A Ertan H. The new wireless EEG device Mentalab Explore is a valid and reliable system for the measurement of resting state EEG spectral features. Brain Res. (2023) 1798:148164. doi: 10.1016/j.brainres.2022.148164, 36402176

[35] Hernández-Chávez A Hamui-Sutton L Muñoz-Comonfort A Sampieri-Cabrera R. Effect of the use of electronic media on the cognitive intelligence, attention, and academic trajectory of medical students. Cureus. (2025) 17:e79513. doi: 10.7759/cureus.79513, 40135005 PMC11936385

[36] Yi L Lv Z-H Hu S-Y Liu Y-Q Yan J-B Zhang H . Validating the accuracy of a multifunctional smartwatch sphygmomanometer to monitor blood pressure. J Geriatr Cardiol. (2022) 19:843. doi: 10.11909/j.issn.1671-5411.2022.11.004 36561062 PMC9748271

[37] Zhang W Zhou Y-N Zhou Y Wang J-G. Validation of the Watch-type HUAWEI WATCH D oscillometric wrist blood pressure monitor in adult Chinese. Blood Press Monit. (2022) 27:353. doi: 10.1097/MBP.0000000000000608 35687029

[38] Matthews CG Klove K. Instruction manual for the adult neuropsychology test battery. Madison, WI: University of Wisconsin Medical School (1964).

[39] Lafayette Instrument Company Grooved pegboard test: user instructions model 32025. Lafayette, USA: Lafayette Instrument Company, Inc. (2002)

[40] Bezdicek O Nikolai T Hoskovcová M Štochl J Brožová H Dušek P . Grooved pegboard predicates more of cognitive than motor involvement in Parkinson’s disease. Assessment. (2014) 21:723–30. doi: 10.1177/1073191114524271, 24590077

[41] Ashendorf L Vanderslice-Barr JL McCaffrey RJ. Motor tests and cognition in healthy older adults. Appl Neuropsychol. (2009) 16:171–6. doi: 10.1080/09084280903098562, 20183169

[42] Strcnge H. Seelhorst U. Kiel U. Correlation between tests of attention and performance on grooved and Purdue pegboards in Normal subjects. Percept Motor Skills. (2002) 95:507–14. doi: 10.2466/pms.2002.95.2.507 12434843

[43] Delorme A Makeig S. EEGLAB: an open source toolbox for analysis of single-trial EEG dynamics including independent component analysis. J Neurosci Methods. (2004) 134:9–21. doi: 10.1016/j.jneumeth.2003.10.009, 15102499

[44] Oostenveld R Fries P Maris E Schoffelen J-M. FieldTrip: open source software for advanced analysis of MEG, EEG, and invasive electrophysiological data. Comput Intell Neurosci. (2011) 2011:1–9. doi: 10.1155/2011/156869, 21253357 PMC3021840

[45] Lee C Kwon J Kim G Hong J Shin D Lee D. A study on EEG based concentration power index transmission and brain computer interface application. J Inst Electron Eng Korea. (2009) 46:41–6. doi: 10.1007/978-3-642-03882-2_142

[46] Sterman MB. Sensorimotor EEG operant conditioning: experimental and clinical effects. Pavlov J Biol Sci. (1977) 12:63–92. doi: 10.1007/BF03004496, 198727

[47] Pritchard C Sculpher M. Productivity costs: principles and practice in economic evaluation. London: Office of Health Economics (2000) isbn:1-899040-76-5.

[48] Krol M Brouwer WBF Severens JL Kaper J Evers SMAA. Productivity cost calculations in health economic evaluations: correcting for compensation mechanisms and multiplier effects. Soc Sci Med. (2012) 75:1981–8. doi: 10.1016/j.socscimed.2012.07.012, 22925428

[49] Department of Human Resources and Social Security of Sichuan Province. Human resource market wage levels and industry labor cost information in the Sichuan–Chongqing region (2024). Available online at: https://rst.sc.gov.cn/rst/zdmsxxssqk/2025/9/3/519b7ff9c0924ed48abd4f084c9f6b01.shtml (Accessed December 6, 2025).

[50] Kim H Hong T Kim J Yeom S. A psychophysiological effect of indoor thermal condition on college students’ learning performance through EEG measurement. Build Environ. (2020) 184:107223. doi: 10.1016/j.buildenv.2020.107223

[51] Abbasi AM Darvishi E Sayehmiri K. Exploring gender differences in acoustic-thermal comfort and performance in a simulated working environment. Build Environ. (2024) 265:111995. doi: 10.1016/j.buildenv.2024.111995

[52] Yang L Zhao S Gao S Zhang H Arens E Zhai Y. Gender differences in metabolic rates and thermal comfort in sedentary young males and females at various temperatures. Energ Build. (2021) 251:111360. doi: 10.1016/j.enbuild.2021.111360

[53] Seppänen O. Fisk W.J. Lei Q. Effect of temperature on task performance in office environment. (2006). Available online at: https://indoor.lbl.gov/publications/effect-temperature-task-performance (Accessed December 6, 2025).

[54] Puyana-Romero V Díaz-Márquez AM Garzón C Ciaburro G. The domestic acoustic environment in online education—part 1: differences by gender, perceived academic quality, and self-rated performance. Buildings. (2024) 15:84. doi: 10.3390/buildings15010084

[55] Allen JG MacNaughton P Satish U Santanam S Vallarino J Spengler JD. Associations of cognitive function scores with carbon dioxide, ventilation, and volatile organic compound exposures in office workers: a controlled exposure study of green and conventional office environments. Environ Health Perspect. (2016) 124:805–12. doi: 10.1289/ehp.1510037, 26502459 PMC4892924

