# Supplementary material for: EEG response and productivity outcome under changing indoor environment
Source: Front Public Health. 2026 Mar 5;14:1767357. doi: 10.3389/fpubh.2026.1767357 (PMC12999586; doi:10.3389/fpubh.2026.1767357)
Supplement: Supplementary file 1 [file Data_Sheet_1.docx]

**Supplemental**

**Table S1.** Specifications and accuracy of instruments.

| **Factors** | **Model** | **Accuracy** |
| --- | --- | --- |
| Temperature | DELIXI ELECTRIC DI-404A | ± 1 °C |
| Acoustics | DELIXI ELECTRIC DSM D2 | ± 1.5 dB |
| Luminescence | DELIXI ELECTRIC EMC L1 | ± (3%+5) lx |
| Relative Humidity | DELIXI ELECTRIC DI-404A | ± 5% RH |
| CO_2_ Concentration | DELIXI ELECTRIC DI-404A | ± (40+3%) ppm |


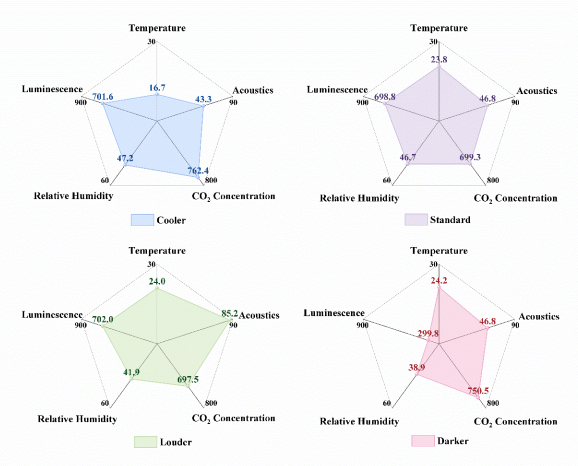


**Figure S1.** Radar chart of monitored environmental factors in four scenarios.

**Table S2.** Results of multicollinearity test.

| **Variable** | **VIF** | **1/VIF** |
| --- | --- | --- |
| Relative Humidity | 1.410 | 0.709 |
| Luminescence | 1.330 | 0.754 |
| Acoustics | 1.230 | 0.810 |
| CO_2_ Concentration | 1.210 | 0.827 |
| Heart Rate | 1.190 | 0.844 |
| Pulse Pressure | 1.040 | 0.957 |
| Mean VIF | 1.240 |  |


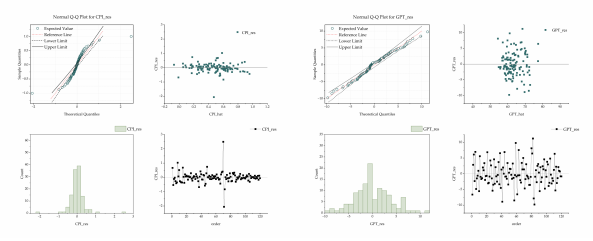


(a) (b)

**Figure S2.** (a) Residual analysis for CPI; (b) Residual analysis for GPT.


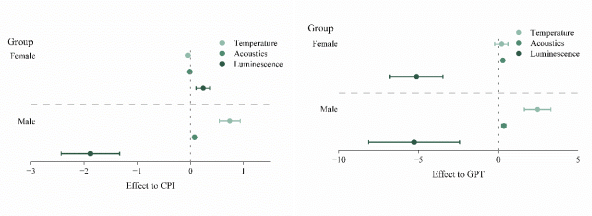


(a) (b)

**Figure S3.** (a) Coefficient Plot for Stratified Analysis of CPI by Gender; (b) Coefficient Plot for Stratified Analysis of GPT by Gender.
